# The Melanogenesis Alteration Effects of *Achillea millefolium* L. Essential Oil and Linalyl Acetate: Involvement of Oxidative Stress and the JNK and ERK Signaling Pathways in Melanoma Cells

**DOI:** 10.1371/journal.pone.0095186

**Published:** 2014-04-17

**Authors:** Hsin-Yi Peng, Chih-Chien Lin, Hsun-Yen Wang, Ying Shih, Su-Tze Chou

**Affiliations:** 1 Department of Food and Nutrition, Providence University, Taichung, Taiwan; 2 Department of Cosmetic Science, Providence University, Taichung, Taiwan; 3 Tri-Service General Hospital, Taipei, Taiwan; The University of Tokyo, Japan

## Abstract

The mitogen-activated protein kinase (MAPK) family, including extracellular signal-regulated kinase (ERK)1/2, c-Jun N-terminal kinase (JNK)1/2 and p38 MAPK, is known to be activated by ultraviolet (UV) radiation in melanocytes to regulate melanin production. Reactive oxygen species (ROS) play important roles in the pathway of ERK and JNK activation. It has been established that the essential oil of *Achillea millefolium* L. (AM-EO) has activities that suppress the oxidative stress and inflammatory responses. Thus, we analyzed the effects of AM-EO on melanogenesis in melanocyte stimulating hormone (α-MSH) treated melanoma cells. The results demonstrated that AM-EO suppresses melanin production by decreasing tyrosinase activity through the regulation of the JNK and ERK signaling pathways. This effect might be associated with the AM-EO activity leading to the suppression of ROS, and linalyl acetate is its major functional component. Therefore, we propose that AM-EO has the potential to treat hyperpigmentation in the future.

## Introduction

Melanin is the primary determinant of skin, hair and eye color. In addition to defining major human phenotypic traits, melanin has an important role in photo-protection because of its capability for ultraviolet (UV) radiation absorption [1]. Melanogenesis is a complex process that is regulated by tyrosinase and tyrosinase-related proteins (TRPs). Tyrosinase plays a critical role in altering melanin production by the hydroxylation of tyrosine into dihydroxyphenylalanine (DOPA) followed by further oxidation of DOPA into DOPA quinone. Therefore, inhibition of tyrosinase is the simplest approach to achieving skin hypopigmentation because it is the key enzyme that catalyzes the rate-limiting step of melanogenesis. In addition, tyrosinase and TRPs are transcriptionally regulated by a key transcription factor in melanocytes, microphthalmia-associated transcription factor (MITF) [2].

Melanocortin receptors belong to the family of G-protein receptors in melanocytes and agonists include melanocyte stimulating hormone (α-MSH), which activates the adenylate cyclase enzyme, increasing intracellular cyclic adenosine monophosphate (cAMP) and activating cAMP response element-binding protein (CREB). CREB acts as a transcription factor for several genes, including MITF [3]. Moreover, mitogen-activated protein kinase (MAPK) family members, including extracellular signal-regulated kinase (ERK)1/2, c-Jun N-terminal kinase (JNK)1/2 and p38 MAPK, are known to be activated by various extracellular stimuli. UV radiation is one such typical extracellular stimulus that induces MAPK activation. Recent studies have demonstrated that MAPKs are implicated in mammalian melanogenesis [4]. Moreover, reactive oxygen species (ROS) also play important roles in the pathways of ERK and JNK activation [5], [6], which are also effective modulators of the activation of MITF, thereby leading to the regulation of melanogenesis.

The most important member of the genus *Achillea* of the Asteraceae family with biological and pharmacological significance is *Achillea millefolium* L., which is commonly known as yarrow [7]. *A. millefolium* has been traditionally used to treat inflammatory and spasmodic gastrointestinal disorders, hepatobiliary conditions and overactive cardiovascular and respiratory ailments [8], [9]. Moreover, our previous study established that *A. millefolium* essential oil (AM-EO) can suppress the oxidative stress and inflammatory responses of lipopolysaccharide (LPS)-stimulated RAW 264.7 macrophages through down-regulation of inducible nitric oxide synthase (iNOS), cyclooxygenase-2 (COX-2), tumor necrosis factor-α (TNF-α) and interleukin-6 (IL-6) expression. The major components of AM-EO are artemisia ketone (14.92%), camphor (11.64%), linalyl acetate (11.51%) and 1,8-cineole (10.15%) [10]. Therefore, AM-EO is a potential candidate for the treatment of oxidative stress mediated disorders, such as UV ray– and environmental factor–induced hyperpigmentation.

In this study, we analyzed the effects of AM-EO on the regulation of melanogenesis in α-MSH treated B16 melanoma cells. In addition, the effects of AM-EO on the repression of ROS in α-MSH treated B16 melanoma cells were examined. Moreover, the involvement of the JNK, p38 and ERK signaling pathways in AM-EO effects on melanogenesis were investigated through MAPK inhibition experiments. We also tested the effects of commercially available AM-EO major components on melanogenesis to elucidate its possible functional constituents.

## Materials and Methods

### Essential oil and cell line

Steam-distilled *Achillea millefolium* L. essential oil (AM-EO) was purchased from Australian Botanical Products, Pty Ltd. (Hallam, Victoria, Australia).Linalyl acetate and camphor were purchased from Sigma-Aldrich Chemicals Co. (St. Louis, MO, USA). The 1,8 cineole was purchased from Alfa Aesar (Ward Hill, MA, USA). The B16 murine melanoma cell line (BCRC 60031) was purchased from the Bioresource Collection and Research Center (BCRC, Hsinchu, Taiwan).

### Materials

The α-melanocyte stimulating hormone (α-MSH), dimethyl sulfoxide (DMSO), monobromobimane (MbBr), phenylmethylsulfonyl fluoride (PMSF), pyrogallol, nitro blue tetrazolium (NBT), nicotinamide adenine dinucleotide phosphate (NADPH), hydrogen peroxide solution (H_2_O_2_), bovine serum albumin (BSA), SP600125 (JNK inhibitor) and PD98059 (ERK inhibitor) were purchased from Sigma-Aldrich Chemicals Co. (St. Louis, MO, USA). The L-3,4-dihydroxyphenylalanine (L-DOPA) that was used in this study was purchased from Merck (Darmstadt, Germany). Dulbecco's modified eagle medium (DMEM), fetal bovine serum (FBS), L-glutamine and penicillin-streptomycin were purchased from Invitrogen Life Technologies (Carlsbad, CA, USA). The goat anti-tyrosinase antibody, rabbit anti-β-actin antibody, rabbit anti-phospho-JNK antibody, mouse anti-phospho-p38 antibody and rabbit anti-phospho-ERK antibody were purchased from Santa Cruz Biotechnology (CA, USA). The enhanced chemiluminescence (ECL) kit was purchased from Amersham Biosciences (NJ, USA). All of the other chemicals that were used were of at least reagent-grade quality. Deionized distilled water (ddH_2_O) for solutions and buffers was purified using a Milli-Q system (Millipore, Bedford, MA, USA).

### Cell culture and cell viability assay

The B16 cells were cultured in DMEM supplemented with 10% FBS, 2 mM glutamine, 100 mg/mL streptomycin and 100 U/mL penicillin. The cells were maintained in a humidified 5% CO_2_ incubator at 37°C and were sub-cultured every 3 to 4 days to maintain logarithmic growth. For cell viability, cells were seeded in a 96-well plate at a density of 5×10^3^ cells/well. After 24 h incubation, different concentrations of samples and 1 nM α-MSH were added to each well of this plate and the plate was then incubated for an additional 72 h. Cell viability was determined by MTT assay.

### Melanin content assay

The melanin content assay from Chen *et al*. was used for this study [11]. Cells were incubated with various concentrations of each treatment and were subsequently co-treated with 1 nM α-MSH for 72 h. After this treatment, the cells were dissolved in the 1 M NaOH/10% DMSO solution and incubated at 90°C to solubilize the melanin. The total melanin in each cell suspension was determined by measuring the absorbance of each suspension at 405 nm. The melanin content was calculated by interpolating the results onto a standard curve that was generated by the absorbance of synthetic melanin and correcting for the total protein amounts that are present in the supernatants of cell lysates.

### Cellular tyrosinase activity assay

Cellular tyrosinase activity was assayed based on DOPA oxidase activity using the method from Seo *et al*. [12]. B16 cells were incubated with various concentrations of each treatment and were subsequently co-treated with 1 nM α-MSH for 72 h. At the endpoint of this treatment, the cells were sonicated with phosphate buffer (pH 6.8) containing 1 mM PMSF. The lysates were clarified by 10 min of centrifugation at 10,000×g. Following the protein quantification of each lysate and protein concentration adjustments with lysis buffer, 300 µL of each lysate was mixed with 700 µL of 5 mM L-DOPA. The mixture was incubated for 1 h at 37°C and the absorbance then measured by spectrophotometer at 475 nm.

### Measurement of superoxide anion production, lipid peroxide, glutathione (GSH) levels and superoxide dismutase (SOD), catalase (CAT), glutathione peroxidase (GPx) activities

B16 cells were incubated with various concentrations of essential oil and 1 nM α-MSH for 72 h prior to testing the superoxide anion production, lipid peroxide and GSH levels and SOD, CAT and GPx activities. Superoxide anion measurement was performed based on the NBT assay according to the method of Freire*et al*. [13] and expressed as a percentage of the untreated group. For lipid peroxide level measurement, cells were harvested and sonicated in 1 mL of cell lysis buffer containing 1 mM PMSF to obtain a cell homogenate. The thiobarbituric acid reactive substances (TBARS) method was used to estimate cellular malondialdehyde (MDA) levels with a spectrophotometer by measuring the absorbance at 535 nm [14]. Glutathione concentration was measured using an enzymatic recycling procedure in which GSH is sequentially oxidized by 2-nitrobenzoic acid and reduced by NADPH in the presence of GSH reductase [15]. The protein content of the cell homogenate was determined based on the Biuret reaction using a BCA kit (Pierce, Rockford, IL, USA) with BSA standards [16]. The MDA and GSH levels in cells are expressed as nanomoles per milligram protein. For enzyme activities, cells were harvested and sonicated in 1 mL of cell lysis buffer containing 1 mM PMSF to obtain a cell homogenate. For glutathione peroxidase activity, one unit of GPx was defined as the amount of enzyme that oxidized 1 nM of NADPH per minute based on the absorbance readings obtained at 340 nm [17]. SOD activity was determined by spectrophotometry based on the absorbance readings obtained at 325 nm, which indicate the SOD-mediated decrease in the rate of pyrogallol autoxidation under alkaline conditions [18]. A unit of SOD activity was defined as the amount of enzyme that inhibited the rate of pyrogallol oxidation. Catalase activity was analyzed by following the decrease in absorbance of H_2_O_2_ at 240 nm. One unit of catalase was defined as the amount of enzyme that decomposed 1.0 µM of H_2_O_2_ per minute [19]. The GPx, SOD and CAT specific activities are expressed as units/mg protein.

### Western blot assay

B16 cells were incubated with various concentrations of each treatment and were subsequently co-treated with 1 nM α-MSH for 72 h. After this treatment, the cells were sonicated with phosphate buffer (pH 6.8) containing 1 mM PMSF, the lysates collected and the protein concentrations quantified. For western blotting, each well was loaded with 20 ng of protein and resolved by 12% SDS PAGE then electrotransferred onto a polyvinylidene difluoride (PVDF) membrane using the Bio-Rad Mini-Protean II apparatus (Bio-Rad Laboratories, Carlsbad, CA, USA). The blots were subsequently incubated with goat anti-tyrosinase antibody or rabbit anti-β-actin antibody and the immune complexes visualized using ECL reagent. The bands were scanned and then quantified by measuring the optical densities using VIpro Platinum 1.1 software (Version 12.9; UVItec, UK).

### Statistical analysis

All measurements were performed at least three times with three different preparations. All data are expressed as the mean ± standard deviation (SD). The analysis of variance was performed using SPSS software (version 16.0; SPSS Inc., USA). One-way ANOVA and Scheffe's method were used to determine the differences between the means, and differences with *P*<0.05 were considered to be statistically significant.

## Results and Discussion

### The effects of AM-EO on cell viability, melanin content and cellular tyrosinase activity

**Figure 1:**
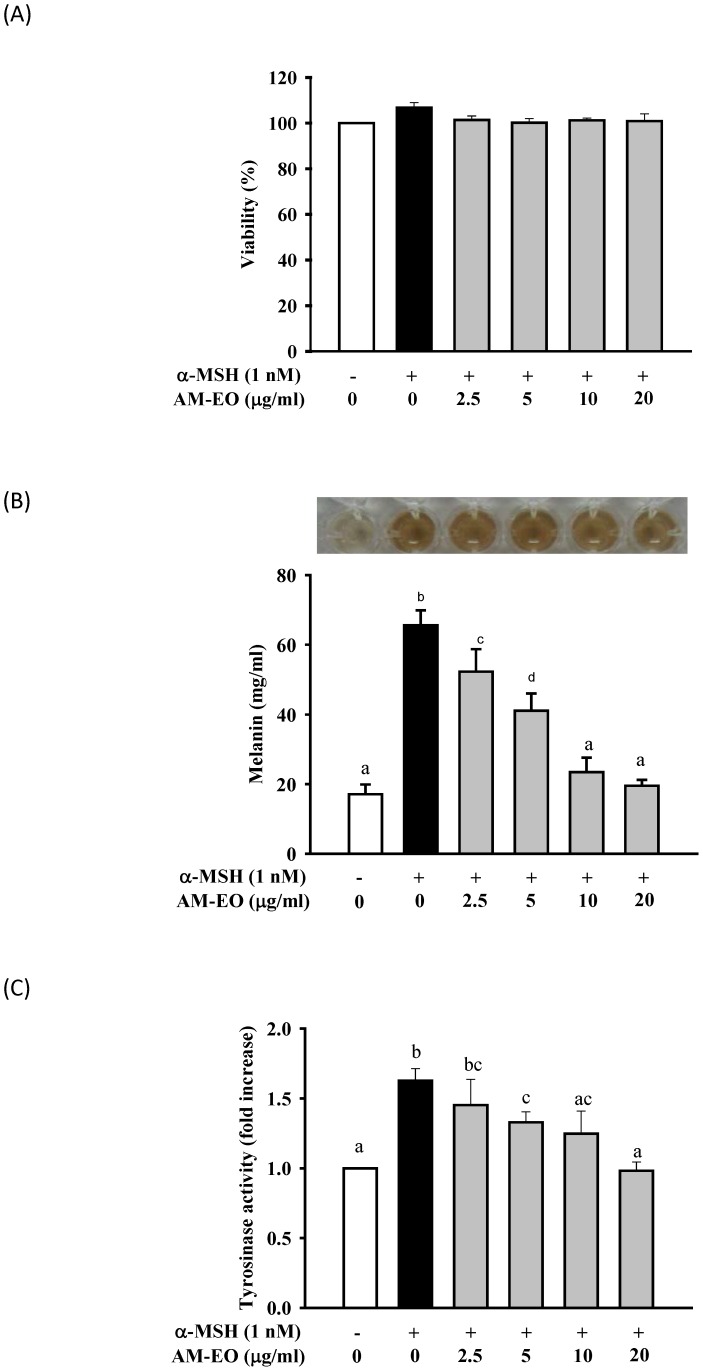
The effects of AM-EO on (A) cell viability, (B) melanin content and (C) cellular tyrosinase activity in α-MSH stimulated B16 cells. Each value represents the mean ± SD (*n* = 3). Groups sharing the same superscript letter are not significantly different (*p*>0.05) as revealed by Dunnett's *post hoc* tests.

We first tested the cytotoxicity of AM-EO on α-MSH stimulated B16 cells and the resultsare shown in Fig. 1A. No significant differences were observed in the viability of B16 cellstreated with AM-EO concentrations between 2.5 and 20 µg/mL and the untreated control. Thus, we used these AM-EO samples for the following experiments.

For the melanin content assay, results are shown in Figure 1B. Melanin content of 1 nM α-MSH treated B16 cells was increased to approximately 3 times that of untreated B16 cells. In the AM-EO treated groups, results clearly demonstrated that AM-EO treatment decreased the melanin production of α-MSH treated B16 cells in a dose-dependent manner. Moreover, the melanin content of 20 µg/mL AM-EO treated B16 cells was diminished to nearly the same as that of untreated cells (Fig. 1B).

The functional hypopigmentation agents can decrease melanin production in melanocytes through the reduction of cellular tyrosinase activity [20]. Thus, to further confirm the function of AM-EO on melanin production, we analyzed cellular tyrosinase activity in α-MSH stimulated B16 cells with or without AM-EO treatment. The results are shown in Figure 1C. The variations of tyrosinase activity in AM-EO treated B16 cells are quite similar to the results for melanin content (Fig. 1B). In 20 µg/mL AM-EO treated B16 cells, cellular tyrosinase activity is also reduced to the same level as that of untreated cells (Fig. 1C). This suggests that AM-EO affects melanin production in α-MSH stimulated B16 cells by decreasing cellular tyrosinase activity.

### The effects of AM-EO on superoxide anion and malondialdehyde (MDA) production, glutathione (GSH) concentration and glutathione peroxidase (GPx), superoxide dismutase (SOD) and catalase (CAT) activities

**Figure 2:**
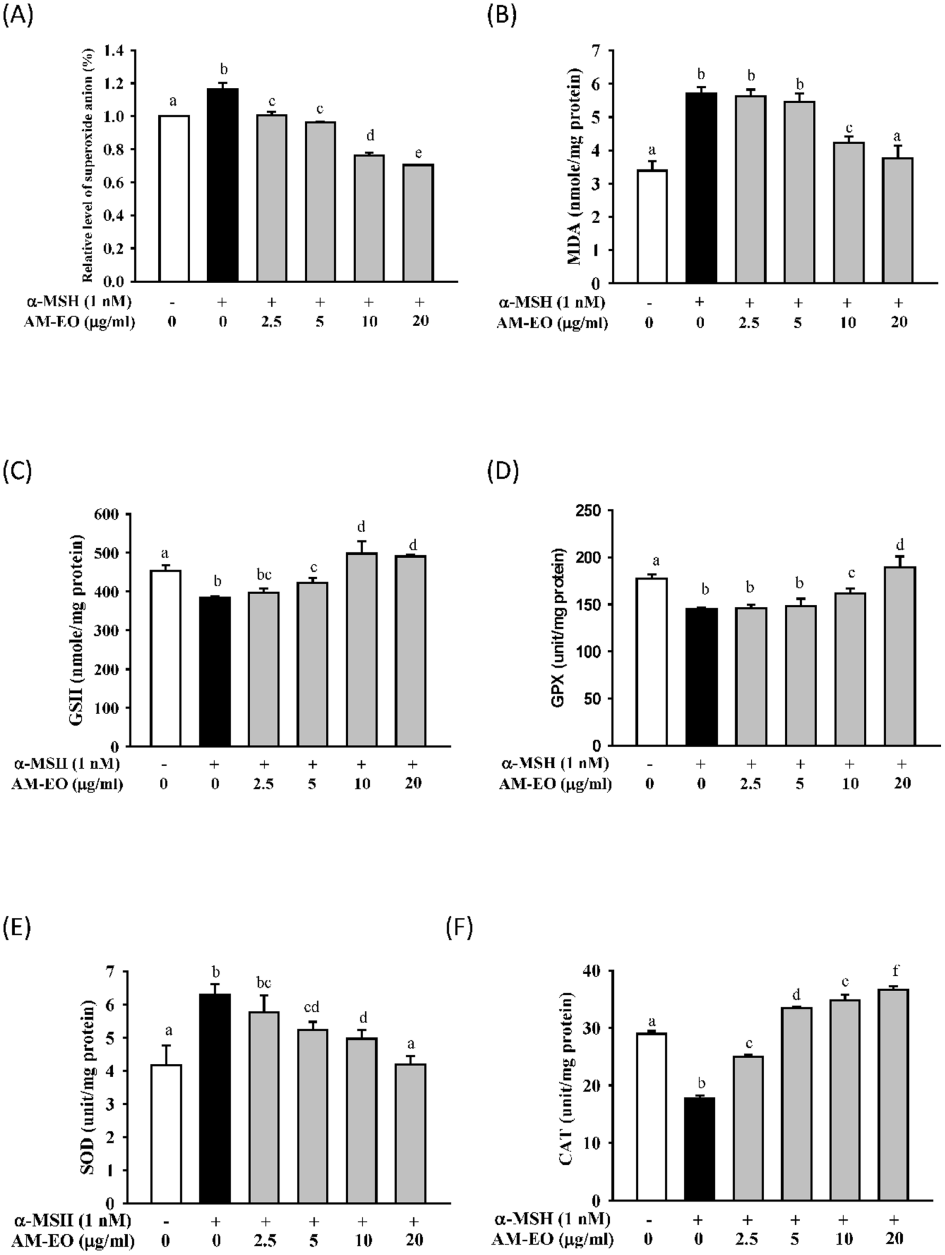
The effects of AM-EO on (A) superoxide anion, (B) malondialdehyde (MDA) production, (C) glutathione (GSH) concentrations and (D) glutathione peroxidase (GPx), (E) superoxide dismutase (SOD) and (F) catalase (CAT) activities in α-MSH stimulated B16 cells. Each value represents the mean ± SD (*n* = 3). Groups sharing the same superscript letter are not significantly different (*p*>0.05) as revealed by Dunnett's *post hoc* tests.

Melanogenesis-mediated reduction of glutathione and cysteine is recognized as an oxidative stress responsein melanocytes. Moreover, in our earlier study, AM-EO was demonstrated to be a potent inflammatory suppressor that inhibits ROS levels in LPS-stimulated macrophages. Thus, we analyzed the effects of AM-EO on the suppression of ROS in α-MSH treated B16 cells; the results are shown in Figure 2.

AM-EO can clearly decrease the α-MSH stimulated superoxide anion level in B16 cells in a dose-dependent manner. The levels of superoxide anion were reduced to only 80% of that in untreated cells in AM-EO concentrations higher than 10 µg/mL (Fig. 2A). In addition, we also tested lipid peroxidation through the production of MDA in α-MSH stimulated B16 cells to verify the effects of AM-EO. The results are shown in Fig. 2B. MDA production in high concentration AM-EO (10 and 20 µg/mL) treated cells was clearly decreased when compared with cells from the control group. This result indicated that AM-EO might successfully repress cellular lipid peroxidation in α-MSH stimulated B16 cells. α-MSH stimulated melanogenesis may consume the GSH in melanocytes. The results shown in Fig. 2C demonstrate that AM-EO treatment can restore cellular GSH levels inα-MSH stimulated B16 cells.

Antioxidant enzymes, including GPx, SOD and CAT, play important roles in sustaining redox homeostasis [21]. Hence, these antioxidant enzymes also respond when cells respond to oxidative stress. As shown in Fig. 2D, the activity of GPx was decreased in α-MSH treated B16 cells. However, the treatment of AM-EO recovered the GPx activity in a dose-dependent manner in α-MSH stimulated B16 cells and this result correlates with that of cellular GSH levels shown in Fig. 2C. As shown in Fig. 2E, α-MSH stimulation increases the activity of SOD to counter oxidative stress. AM-EO treatment clearly reduced the α-MSH increased SOD activity in B16 cells. At a concentration of 20 µg/mL AM-EO, levels of SOD activity were decreased to the same as that of untreated cells (Fig. 2E). α-MSH stimulation decreased the levels of CAT activity in B16 cells; however, treatment with AM-EO restored CAT activity levels in α-MSH treated cells at concentrations higher than 5 µg/mL (Fig. 2F). MC1R stimulation by α-MSH rapidly induces catalase expression through a cAMP/PKA dependent and MITF independent mechanism at the post-transcriptional level; however, this stimulation of catalase is observed only within 48 h [22]. Consequently, in our experiment, CAT activities were examined after 72 h of treatment. This is a rational explanation for the observation that α-MSH stimulation decreased the level of CAT activity in B16 cells in this study. In addition, although these results are also slightly different from our experiments in RAW 264.7 macrophages [10], we can still confirm that the effects of AM-EO on melanogenesis alteration might be associated with its function inoxidative stress suppression.

### The effects of AM-EO on tyrosinase, p-JNK, p-p38 and p-ERK protein levels

**Figure 3:**
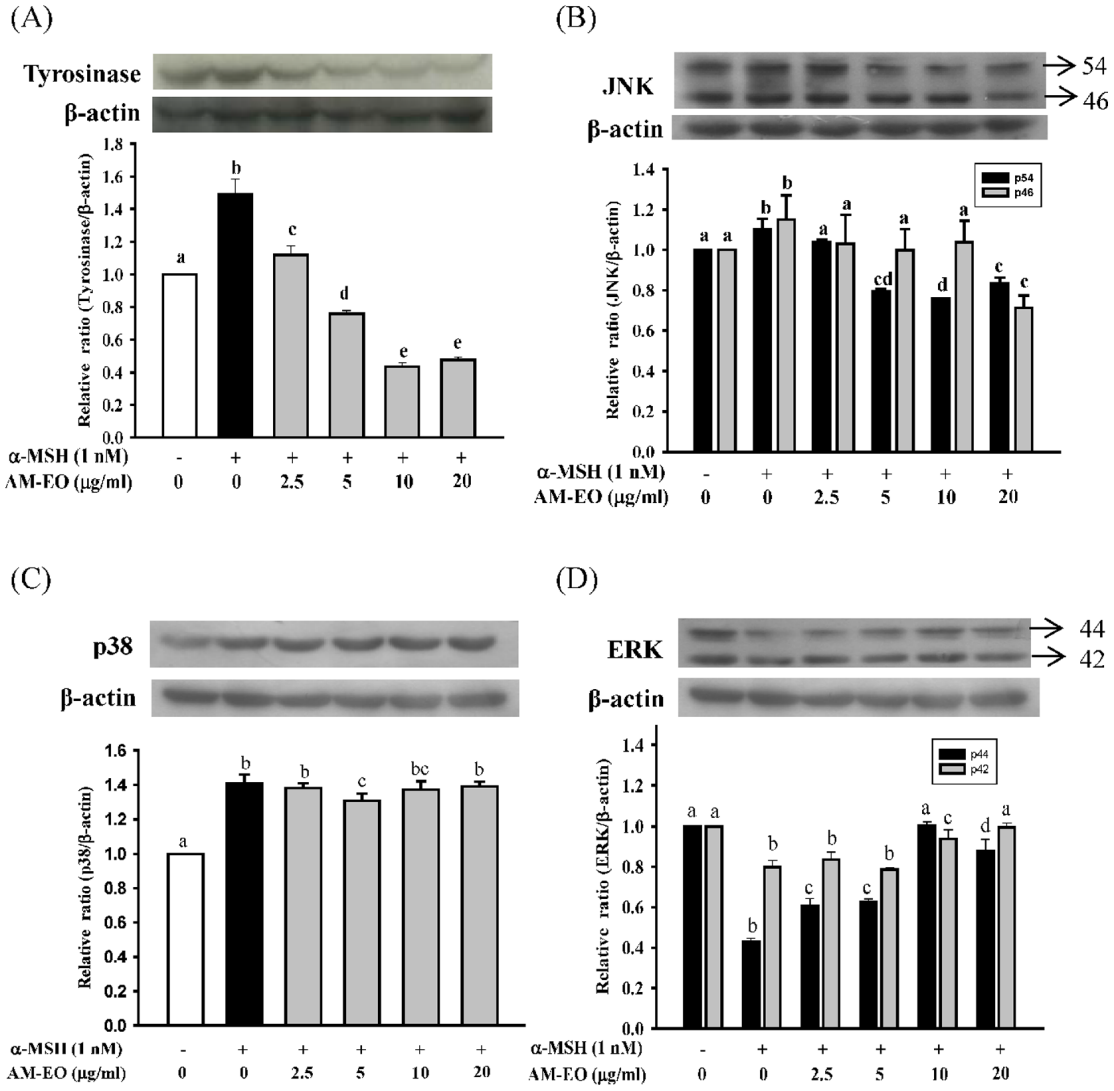
The effects of AM-EO on protein levels of (A) tyrosinase, (B) p-JNK, (C) p-p38 and (D) p-ERK in α-MSH stimulated B16 cells. Each value represents the mean ± SD (*n* = 3). Groups sharing the same superscript letter are not significantly different (*p*>0.05) as revealed by Dunnett's *post hoc* tests. For p-JNK, p54 and p46 are p-JNK1 and p-JNK2, respectively. For p-ERK, p44 and p42 are p-ERK1 and p-ERK2, respectively.

Because UV radiation is a typical extracellular stimulus that induces MAPK activation, to further understand the effect of AM-EO on melanogenesis alteration, we examined the protein levels of tyrosinase, p-JNK, p-p38 and p-ERK in α-MSH stimulated B16 cells. The resultsare shown in Fig. 3. The expression of tyrosinase was induced to 1.5 times that of the control group upon treatment with α-MSH in B16 cells. The AM-EO treatment obviously down-regulated the induction of tyrosinase expression in α-MSH stimulated B16 cells. Furthermore, if the concentrations of AM-EO were higher than 5 µg/mL, the repression of tyrosinase expression was diminished to levels lower than those of the control group (Fig. 3A). This result is also similar to those for melanin content and tyrosinase activity in Fig. 1B and 1C.

Our results demonstrated that α-MSH stimulation increased the levels of p-JNK1 (p54), pJNK2 (p46) and p-p38 in B16 cells (Fig. 3B and 3C). However, higher concentrations of AM-EO only suppressed the levels of p-JNK1 and pJNK2 (Fig. 3B) but not the levels of p-p38 (Fig. 3C). Moreover, stimulation with α-MSH reduced the levels of p-ERK1 (p54) and pERK2 (p46) in B16 cells (Fig. 3D). The amounts of p-ERK1 (p54) and pERK2 (p46) were restored to normal levels by treatment with higher concentrationsof AM-EO (10 and 20 µg/mL). Therefore, these results indicate that MAPKs are involved in melanogenesis in B16 cells. Furthermore, AM-EO reduced the phosphorylation of JNK and enhanced the phosphorylation of ERK without affecting the phosphorylation of p38 MAPK. Thus, we concludethat JNK1/2 and ERK1/2 pathways are associated with the effects of AM-EO on melanogenesis alteration in B16 cells.

### The effects of JNK and ERK inhibitors on the expression of tyrosinase

**Figure 4:**
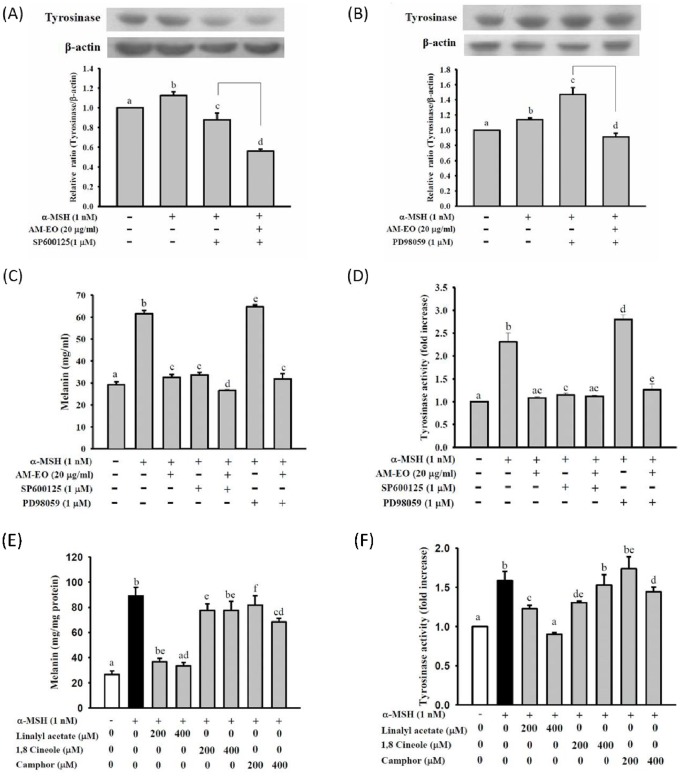
The effects of the JNK inhibitor SP600125 and the ERK inhibitor PD98059 on (A and B) protein levels of tyrosinase, (C) melanin content and (D) cellular tyrosinase activity in α-MSH stimulated B16 cells. The effects of the major components of AM-EO, linalyl acetate, 1,8-cineole and camphor, on (E) melanin content and (F) cellular tyrosinase activity in α-MSH stimulated B16 cells. Each value represents the mean ± SD (*n* = 3). Groups sharing the same superscript letter are not significantly different (*p*>0.05) as revealed by Dunnett's *post hoc* tests.

To further confirm that the JNK and ERK signaling pathways are correlated with the regulation of AM-EO effects on melanogenesis in α-MSH stimulated B16 cells, we used the JNK and ERK inhibitors SP600125 and PD98059 to test the melanogenesis altering effects of AM-EO. As shown in Fig. 4A, the expression of tyrosinase in α-MSH stimulated B16 cells was decreased by the JNK inhibitor SP600125. Co-treatment of cells with 1 µM SP600125 and 20 µg/mL AM-EO suppressed tyrosinase protein levels to less than 60% of the control group (Fig. 4A). In contrast, upon treatment with the ERK inhibitor PD98059, the expression of tyrosinase in α-MSH stimulated B16 cells was up-regulated to approximately 1.5 times that of the control group. Co-treatment with 20 µg/mL AM-EO and1 µM PD98059 clearly suppressed the tyrosinase expression to 91% that of the control group in α-MSH stimulated B16 cells (Fig. 4B).

### The effects of JNK and ERK inhibitors on melanin content and cellular tyrosinase activity

The effects of JNK and ERK inhibitors on melanin content and cellular tyrosinase activity were also analyzed in this study. Treatment with SP600125 and PD98059 respectively decreased and increased the melanin content in α-MSH treated B16 cells. Moreover, in the groups treated with the JNK and ERK inhibitors, the melanin content increase was repressed by the addition of AM-EO (Fig. 4C). Fig. 4D shows that the results for cellular tyrosinase activity are almost the same as the results for melanin content. SP600125 and PD98059 decreased and increased cellular tyrosinase activity in α-MSH treated B16 cells, respectively, and tyrosinase activity was also down-regulated by the addition of AM-EO. Therefore, according to these results, we can presume that AM-EO decreases the production of melanin through the repression of cellular tyrosinase by regulating the JNK and ERK signaling pathways in α-MSH stimulated B16 cells.

Several studies have found that p38 is a key intracellular signaling molecule for pigmentation and that activation of the p38 MAPK pathway has been reported to be related to an increase in melanin synthesis [23]. Moreover, JNK1/2 and ERK1/2 are related to the regulation of melanogenesis through MITF and tyrosinase activities [24], [25], [26]. Our results also demonstrated that AM-EO decreases the production of melanin through the down-regulation of tyrosinase activity and this effect is associated with the regulation of the JNK and ERK signaling pathways in α-MSH stimulated B16 cells.

### The effects of linalyl acetate, 1,8-cineole and camphor on melanin content and cellular tyrosinase activity

Our earlier study confirmed that the major constituents of AM-EO are artemisia ketone (14.92%), camphor (11.64%), linalyl acetate (11.51%) and 1,8-cineole (10.15%). To our knowledge, except for artemisia ketone, the other 3 components are commercial obtainable. Therefore, to further evaluate the functional compounds of AM-EO on the alteration of melanogenesis, we used camphor, linalyl acetate and 1,8-cineole to test the effects on melanin content and cellular tyrosinase activity in α-MSH stimulated B16 cells. The treatment with linalyl acetate obviously repressed the melanin levels at both concentrations used and the repression effect decreased the melanin levels to almost the same as that of the control group (Fig. 4E). Although camphor and 1,8-cineole also have some inhibitory effects on melanin production in α-MSH stimulated B16 cells, the repression effects were not as significant as when compared with those of linalyl acetate. In addition, as shown in Fig.4F, the results of cellular tyrosinase activities were also similar to those of melanin content. Linalyl acetate reduced cellular tyrosinase activity in α-MSH stimulated B16 cells in a dose-dependent manner more effectively than the other compounds (Fig.4F). Therefore, our results demonstrate that linalyl acetate is the major functional component of AM-EO with effects on melanogenesis alteration in α-MSH stimulated B16 cells. Linalyl acetate is found in many plants, including the essential oils of *Citrus bergamia*, *Citrus aurantifolia* and *Citrus aurantium*, as well as in *Achillea millefolium* [10], [27]–[28]. Some studies have reported that linalyl acetate has antioxidant and antimicrobial activities [29];however, to our knowledge, this is the first report that linalyl acetate has an effect on the regulation of melanogenesis.

## Conclusion

**Figure 5:**
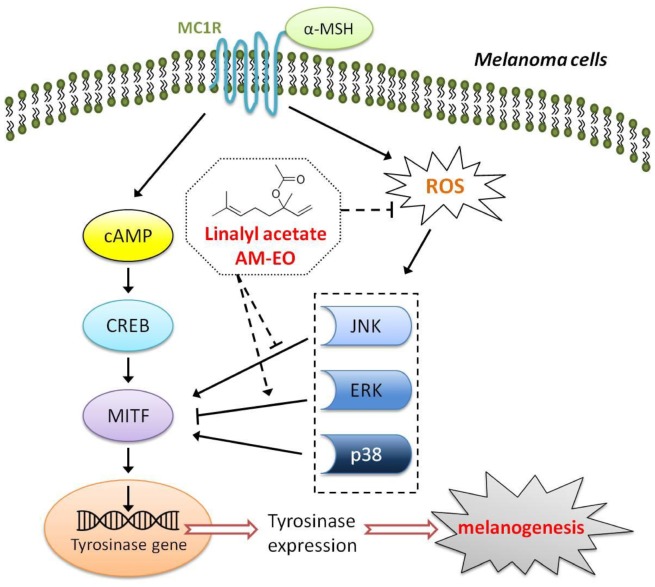
The proposed mechanism of AM-EO and linalyl acetate effects on melanogenesis in melanoma cells.

In summary, as shown in Fig.5, linalyl acetate is the major functional component of AM-EO executing its effects on melanogenesis alteration. AM-EO can suppress the production of melanin by downregulation of tyrosinase activity through the regulation of the JNK and ERK signaling pathways in α-MSH treated melanoma cells. The effects of AM-EO on melanogenesis alteration might be associated with its function in the suppression of ROS. Therefore, we propose that AM-EO has the potential to become an ingredient in hypopigmentation drugs, foods, and cosmetics in the future.

## References

[1] Orazio J, Jarrett S, Amaro-Ortiz A, Scott T (2013) UV radiation and the skin. Int J Mol Sci 14: 12222–12248. 23749111 10.3390/ijms140612222 PMC3709783

[2] Chang TS (2012) Natural melanogenesis inhibitors acting through the down-regulation of tyrosinase activity. Materials 5: 1661–1685.

[3] Videira IF, Moura DF, Magina S (2013) Mechanisms regulating melanogenesis. An Bras Dermatol 88: 76–83. 23539007 10.1590/S0365-05962013000100009 PMC3699939

[4] Muthusamy V, Piva TJ (2010) The UV response of the skin: a review of the MAPK, NF_k_B and TNFα signal transduction pathways. Arch Dermatol Res 302: 5–17. 19756672 10.1007/s00403-009-0994-y

[5] Johnson GL, Nakamura K (2007) The c-jun kinase/stress-activated pathway: regulation, function and role in human disease. Biochim Biophys Acta 1773: 1341–1348. 17306896 10.1016/j.bbamcr.2006.12.009 PMC1995559

[6] Son Y, Cheong YK, Kim NH, Chung HT, Kang DG, et al.. (2011) Mitogen-activated protein kinases and reactive oxygen species: how can ROS activate MAPK pathways? J Signal Transduct doi:10.1155/2011/792639. 10.1155/2011/792639 PMC3100083 21637379

[7] Feizpour A, Boskabady MH, Byrami G, Golamnezhad Z, Shafei MN (2013) The effect of hydro-ethanolic extract of *Achillea millefolium* on muscarinic receptors of guinea pig tracheal smooth muscle. Indian J Pharmacol 45: 13–17. 23543621 10.4103/0253-7613.106428 PMC3608287

[8] Benedek B, Kopp B (2007) *Achillea millefolium* L. s.l. revisited: recent findings confirm the traditional use. Wien Med Wochenschr 157: 312–314. 17704978 10.1007/s10354-007-0431-9

[9] Khan AU, Gilani AH (2011) Blood pressure lowering, cardiovascular inhibitory and bronchodilatory actions of *Achillea millefolium* . Phytother Res 25: 577–583. 20857434 10.1002/ptr.3303

[10] Chou ST, Peng HY, Hsu JC, Lin CC, Shih Y (2013) *Achillea millefolium* L. essential oil inhibits LPS-induced oxidative stress and nitric oxide production in RAW 264.7 macrophages. Int J Mol Sci 14: 12978–12993. 23797659 10.3390/ijms140712978 PMC3742169

[11] Chen YS, Lee SM, Lin CC, Liu CY, Wu MC, et al (2013) Kinetic study on the tyrosinase and melanin formation inhibitory activities of carthamus yellow isolated from *Carthamus tinctorius* L. J Biosci Bioeng 115: 242–245. 23063243 10.1016/j.jbiosc.2012.09.013

[12] Seo WD, Ryu YB, Curtis-Long MJ, LeeCW, Ryu HW, et al (2010) Evaluation of anti-pigmentary effect of synthetic sulfonylamino chalcone. Eur J Med Chem 45: 2010–2017. 20149498 10.1016/j.ejmech.2010.01.049

[13] Freire AC, de Assis CF, Frick AO, da Silva Melo P, Haun M, et al (2003) Influence of protein phosphatase inhibitors on HL60 cells death induction by dehydrocrotonin. Leuk Res 27: 823–829. 12804641 10.1016/s0145-2126(03)00013-4

[14] Huang CS, Hu HH, Tsai YM, Chang WT (2013) In vitro effects of *Monascus purpureus* on antioxidation activity during fermentation of Kinmen sorghum liquor waste. J Biosci Bioeng 115: 418–423. 23266115 10.1016/j.jbiosc.2012.11.003

[15] Park CM, Park JY, Noh KH, Shin JH, Song YS (2011) *Taraxacum officinale* Weber extracts inhibit LPS-induced oxidative stress and nitric oxide production via the NF-_k_B modulation in RAW 264.7 cells. J Ethnopharmacol 133: 834–842. 21075189 10.1016/j.jep.2010.11.015

[16] Smith PK, Krohn RI, Hermanson GT, Mallia AK, Gartner FH, et al (1985) Measurement of protein using bicinchoninic acid. Anal Biochem 150: 76–85. 3843705 10.1016/0003-2697(85)90442-7

[17] Bak MJ, Jun M, Jeong WS (2012) Antioxidant and hepatoprotective effects of the red ginseng essential oil in H_2_O_2_-treated HepG2 cells and CCl_4_-treated mice. Int J Mol Sci 13: 2314–2330. 22408456 10.3390/ijms13022314 PMC3292025

[18] Yin H, Miao J, Ma C, Sun G, Zhang Y (2012) β-Casomorphin-7 cause decreasing in oxidative stress and inhibiting NF-κB-iNOS-NO signal pathway in pancreas of diabetes rats. J Food Sci 77: C278–282. 22339544 10.1111/j.1750-3841.2011.02577.x

[19] Yao X, Zhu L, Chen Y, Tian J, Wang Y (2013) *In vivo* and *in vitro* antioxidant activity and α-glucosidase, α-amylase inhibitory effects of flavonoids from *Cichorium glandulosum* seeds. Food Chem 139: 59–66. 23561078 10.1016/j.foodchem.2012.12.045

[20] Jeong YM, Oh WK, Tran TL, Kim WK, Sung SH, et al (2013) Aglycone of Rh4 inhibits melanin synthesis in B16 melanoma cells: possible involvement of the protein kinase A pathway. Biosci Biotechnol Biochem 77: 119–125. 23291754 10.1271/bbb.120602

[21] Lee BJ, Lin YC, Huang YC, Ko YW, Hsia S, et al.. (2012) The relationship between coenzyme Q10, oxidative stress, and antioxidant enzymes activities and coronary artery disease. Scientific World Journal doi.org/10.1100/2012/792756. 10.1100/2012/792756 PMC3356738 22645453

[22] Maresca V, Flori E, Bellei B, Aspite N, Kovacs D, et al (2010) MC1R stimulation by α-MSH induces catalase and promotes its re-distribution to the cell periphery and dendrites. Pigment Cell Melanoma Res 23: 263–275. 20067588 10.1111/j.1755-148X.2010.00673.x

[23] Hirata N, Naruto S, Ohguchi K, Akao Y, Nozawa Y, et al (2007) Mechanism of the melanogenesis stimulation activity of (-)-cubebin in murine B16 melanoma cells. Bioorg Med Chem 15: 4897–4902. 17521910 10.1016/j.bmc.2007.04.046

[24] Kim KN, Yang HM, Kang SM, Kim D, Ahn G, et al (2013) Octaphlorethol A isolated from *Ishige foliacea* inhibits α-MSH-stimulated induced melanogenesis via ERK pathway in B16F10 melanoma cells. Food Chem Toxicol 59: 521–526. 23810793 10.1016/j.fct.2013.06.031

[25] Yen FL, Wang MC, Liang CJ, Ko HH, Lee CW (2012) Melanogenesis inhibitor(s) from *Phyla nodiflora* extract. Evid Based Complement Alternat Med 2012: 867494. 23304221 10.1155/2012/867494 PMC3524650

[26] Zhao LM, Han LN, Ren FZ, Chen SH, Liu LH, et al (2012) An ester extract of *Cochinchina momordica* seeds induces differentiation of melanoma B16 F1 cells via MAPKs signaling. Asian Pac J Cancer Prev13: 3795–3802. 10.7314/apjcp.2012.13.8.3795 23098473

[27] Furneri PM, Mondello L, Mandalari G, Paolino D, Dugo P, et al (2012) *In vitro* antimycoplasmal activity of *Citrus bergamia* essential oil and its major components. Eur J Med Chem 52: 66–69. 22465092 10.1016/j.ejmech.2012.03.005

[28] Tundis R, Loizzo MR, Bonesi M, Menichini F, Mastellone V, et al. (2012) Comparative study on the antioxidant capacity and cholinesterase inhibitory activity of *Citrus aurantifolia* Swingle, C. *aurantium* L., and C. *bergamia* Risso and Poit. peel essential oils. J Food Sci 77: ; H40–46. 10.1111/j.1750-3841.2011.02511.x 22260108

[29] Ellouze I, Abderrabba M, Sabaou N, Mathieu F, Lebrihi A, et al (2012) Season's variation impact on *Citrus aurantium* leaves essential oil: chemical composition and biological activities. J Food Sci 77: T173–180. 22897411 10.1111/j.1750-3841.2012.02846.x

